# Social Hygiene

**Published:** 1915-03

**Authors:** 


### Social Hygiene.

"Social Hygiene" has just made its debut as the organ of the American Social Hygiene Association. As such it has a special mission. This association has correlated the various interests on the great question of social reform and is making a heroic effort to accomplish definite results. It will speak to the public through this quarterly and by authentic information hopes to bring about a better understanding and co-operation in the work of social betterment.

The present volume is particularly valuable, giving a score or more original papers by the very best observers on this question; besides it contains a comprehensive bibliography of the books now published on the subjects, and also reviews of some of the more recent books. In our next issue we will endeavor to give a resumé of the more salient questions presented.

